# Feline immunodeficiency virus-mediated long-term transgene expression in undifferentiated retinal progenitor cells and its downregulation in differentiated cells

**Published:** 2008-11-26

**Authors:** Branislava Janic, Xuxiang Zhang, Wei Li

**Affiliations:** 1 Bascom Palmer Eye Institute, Department of Ophthalmology, University of Miami School of Medicine, Miami, FL; 2 Department of Ophthalmology, Xuanwu Hospital, Capital Medical University, Beijing, China; 3 Department of Microbiology and Immunology, University of Miami School of Medicine, Miami, FL

## Abstract

**Purpose:**

Lentivirus-mediated gene transfer is an important approach to modify the function of progenitor cells in ex vivo gene therapy, but may be susceptible to downregulation due to transcriptional silencing. The purpose of this study was to analyze the stability of lentivirus-mediated transgene expression in undifferentiated and differentiated retinal progenitor cells (RPCs), and to characterize the effect of lentivirus transduction on RPC differentiation in vitro.

**Methods:**

RPCs derived from postnatal day 1 mice were expanded in defined serum-free culture medium and transduced with nonprimate lentiviral vector of feline immunodeficiency virus (FIV) expressing yellow fluorescent protein (YFP) reporter. Long-term expression of YFP in undifferentiated and differentiated RPCs was analyzed. Expression of various markers for RPCs and differentiated cells was analyzed by immunochemical staining in lentivirus-transduced and control RPCs. Differentiated postmitotic cells were revealed by negative labeling of bromodeoxyuridine (BrdU).

**Results:**

FIV transduction induced long-term expression of YFP reporter in RPCs for up to 53 days (10 passages) with no sign of decrease in expression level. FIV transduction did not alter the expression profile of various markers in retinal spheres, including nestin, microtubule-associated protein 2 (MAP-2), glial fibrillary acidic protein (GFAP), and opsin. However, YFP expression was downregulated in differentiated BrdU-negative postmitotic cells.

**Conclusions:**

FIV-mediated long-term expression of transgene in undifferentiated RPCs is downregulated upon their differentiation. Thus, lentivirus-mediated ex vivo modulation should be cautiously analyzed for transgene expression not only in undifferentiated RPCs, but also in differentiated postmitotic cells.

## Introduction

Retinal progenitor cells (RPCs) are multipotent precursors that can give rise to different types of retinal cells and thus hold the potential to be used to treat degenerative retinal diseases by cell replacement therapy [1-4]. RPCs are typically isolated from the retina or ciliary margin and have the ability to maintain their proliferative capacity in vitro. RPCs have many similar characteristics to neural progenitor cells (NPCs). Both cell types can grow in the same culture conditions supplemented with growth factors, form clonal spheres with similar morphology, and express the progenitor marker nestin. However, RPCs isolated from the ciliary margin are independent of exogenous basic fibroblast growth factor (bFGF) by supplementing their own bFGF in an autocrine fashion [5]. RPCs have the capacity to differentiate into unique cell lineages expressing retina-specific markers, such as opsin for photoreceptors.

Genetic engineering of progenitor cells with viral vectors followed by in vivo transplantation (ex vivo gene therapy) has multiple potential applications, including delivery of therapeutic proteins and modulation of progenitor cell differentiation and function [6]. One of the challenges for gene transfer with lentiviral or retroviral vectors is potential loss of transgene expression after transplantation [7-9], even though the transplanted cells may survive and integrate well into host tissues. Because previous studies have suggested that lentiviral vectors may be more resistant to stem cell-specific gene silencing in various types of stem cells [10,11], we were interested in the possible silencing of lentivirus-mediated transgene expression. Feline immunodeficiency virus (FIV) is of particularly interest because of safety concerns [12,13]. Unlike human immunodeficiency virus (HIV)-based lentiviral vectors, FIV vectors are derived from a nonhuman pathogen. Routine exposure to FIV fails to induce seroconversion or disease in humans. A legitimate concern for the use of HIV vectors in human subjects is the potential for vector mobilization following HIV infection. However, the mobilization of a second or third generation of FIV-based vectors by HIV gag and pol proteins has not been detected [13]. This lack of significant cross-packaging of FIV vectors by HIV makes FIV vectors attractive vehicles for gene delivery to stem cells, including RPCs. The ability of retroviral and lentiviral vectors to induce stable transgene expression in RPCs has not been defined, and possible downregulation of transgene expression in differentiated RPCs is yet to be characterized. A recent report of transgene silencing by retrovirus- and lentivirus-mediated gene transfer in differentiated NPCs [14] prompted us to examine FIV-mediated long-term transgene expression in RPCs and possible silencing in differentiated cells in this study.

Here we used a second generation FIV vector to drive the expression of yellow fluorescent protein (YFP) in RPCs. Stable transgene expression in FIV-transduced RPCs was demonstrated. However, the transgene expression was downregulated in differentiated bromodeoxyuridine (BrdU)-negative postmitotic cells, suggesting that FIV-mediated transgene expression is also subjected to the transcriptional silencing in RPCs, similar to the HIV-based lentivirus silencing previously reported in NPCs [14].

## Methods

### RPC isolation and expansion

RPCs were isolated from the neural retina of C57BL/6 mice (The Jackson Laboratory, Bar Harbor, ME) at postnatal day 1, as previously described [15]. Animal procedures were conducted in accordance with the National Institutes of Health Animal Care and Use Committee protocols. The periphery of the retina and the optic nerve stalk were removed. Retinal tissue was dissected and digested for 1 h in Dulbecco's Modified Eagle's Medium (DMEM)/F-12 (Invitrogen, Carlsbad, CA) containing 0.1% collagenase (Sigma, St. Louis, MO). Cells were subsequently filtered through a 40 µm nylon mesh (BD Bioscience, Bedford, MA), centrifuged, and resuspended in DMEM/F-12 media supplemented with 10% FBS, 100 µg/ml N-2 neural supplement (Invitrogen), 100 µg/ml penicillin/streptomycin, 2 mM L-glutamine, and 1 µg/ml fungizone. Cells were then incubated at 37 °C. After reaching confluence within a week, cells were trypsinized with 0.1% trypsin-EDTA (Invitrogen), washed with phosphate buffered saline (PBS, resuspended in serum-free DMEM/F-12 media supplemented with N-2, 20 ng/ml epidermal growth factor (EGF) (Sigma) and 20 ng/ml bFGF (Sigma). Cells were incubated at 37 °C until retinal spheres were formed and propagated. RPC spheres were split every 5–7 days with NeuroCult Chemical Dissociation kit (StemCell Technologies, Vancouver, BC, Canada) according to the manufacturer’s protocol with minor modifications. Briefly, the volumes of solutions B and C in the kit were doubled during cell passaging to improve RPC dissociation for clonogenic culture. This modified protocol can efficiently dissociate RPCs with more than 95% viability. Dissociated RPCs were plated at roughly 200 cells/ml for clonogenic expansion and split when the cell density at approximately 20,000 cells/ml (about 5–7 days).

### Lentiviral infection

**Figure 1:**
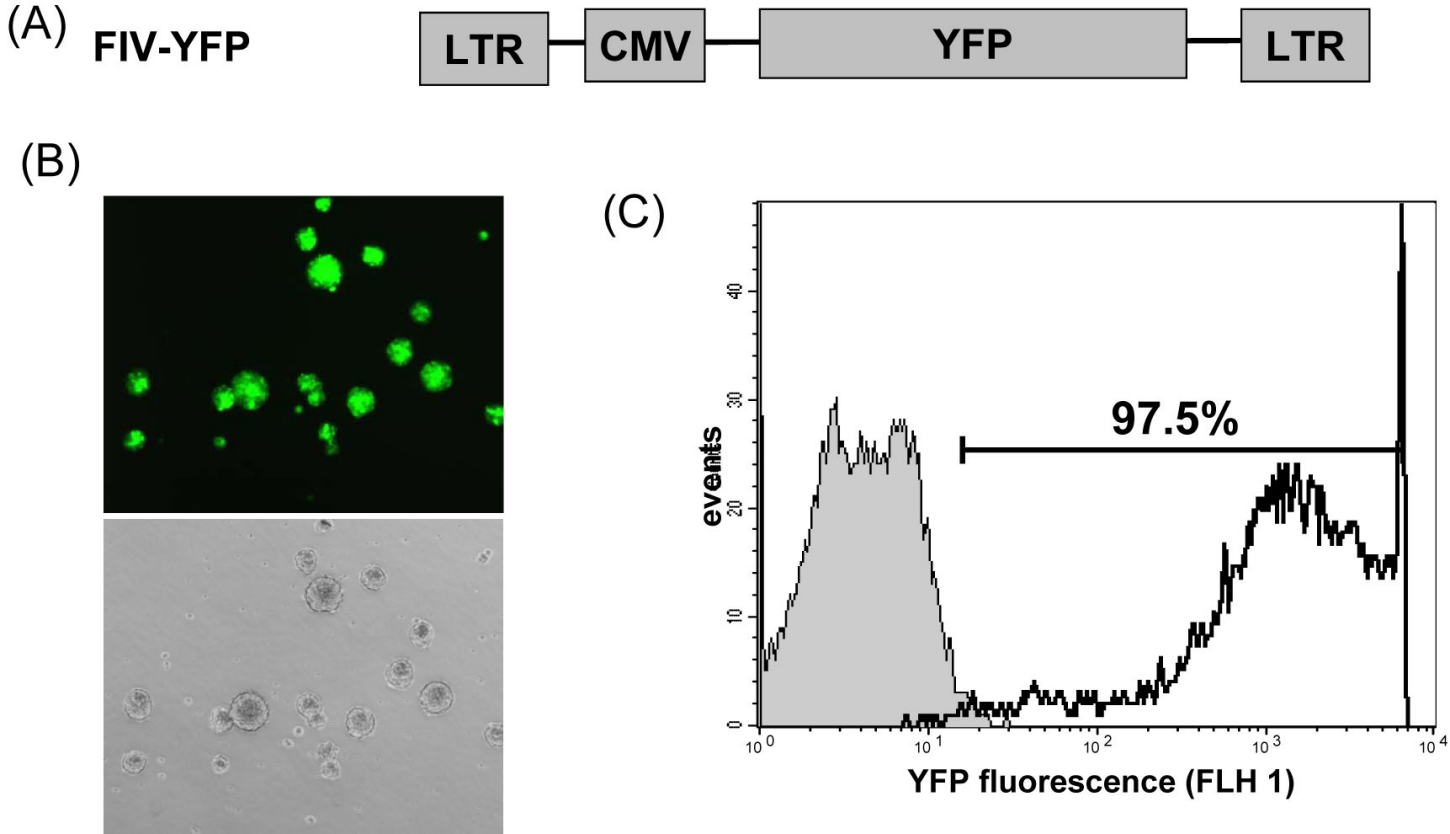
Lentivirus transduction of retinal progenitor cells. **A:** Schematic representation of feline immunodeficiency virus (FIV) vector. Abbreviations are: LTR, FIV long-terminal repeat; CMV, human cytomegalovirus promoter, and YFP, yellow fluorescent protein. **B:** In vitro live fluorescence image of retinal progenitor cells (RPCs) spheres for yellow fluorescent protein (YFP) expression (top, fluorescence field; bottom, bright field). RPCs were transduced with FIV-YFP and analyzed for YFP expression at day 8 post-transduction with an Olympus IX50 inverted fluorescence microscope. **C:** Flow cytometry analysis of YFP expression in RPCs. Flow cytometric histogram of control cells (filled gray histogram) and FIV-YFP transduced cells (open black line) from representative experiment is shown. At least 5,000 gated cells were analyzed for YFP expression. The majority of FIV-transduced RPCs (97.5%) were YFP positive.

Second-generation FIV-based lentiviral vectors were gifts from Dr. Garry Nolan (Stanford University, Stanford, CA) [13,16]. Information for pFIV-YFP (pLionII-YFP; Figure 1A) and other packaging plasmids are available at FELIX. For FIV production, viral supernatants were generated by cotransfecting 293T cells with 3.75 µg pFIV-YFP, 4.5 µg pCI-VSVG, and 6.25 µg pCPRΛEnv plasmids in 100 mm culture plates by calcium phosphate precipitation. FIV-YFP in Opti-MEM medium (Invitrogen) supplemented with 1% FBS was collected every 12 h between 36 h to 60 h posttransfection. FIV titer was determined by infecting fresh 293T cells with FIV-conditioned medium in serial dilutions. FIV-infected yellow fluorescent protein (YFP)-positive cells were analyzed by flow cytometry analysis two days after the infection. A typical titer for FIV-conditioned medium was approximately 1×10^6^ pfu/ml. Before infection, RPCs were dissociated, and the resultant single cell suspension was incubated for 6 h with filtered lentivirus-containing supernatants at approximately 20 multiplicity of infection (MOI) supplemented with 1 μg/ml polybrene (Sigma). YFP expression was analyzed using a Olympus fluorescence IX50 inverted fluorescence microscope (Olympus 100W fluorescence light source and the filter set for Exciter at D480/30x and Emitter at D535/40m; Olympus, Center Valley, PA), and flow cytometry. All the results in this study represented at least three independent experiments starting from FIV infection.

### Flow cytometry analysis

RPCs infected with lentivirus were chemically dissociated as described in the previous section and washed once in PBS. Fluorescence-activated flow cytometry was performed with a FACScan 2000 flow cytometer (Becton Dickinson, Franklin Lakes, NJ). A total of 5,000 events were analyzed for each sample. Live cells were gated for analysis based on forward angle light scatter (FSC) and side angle light scatter (SSC) with laser excitation at 488 nm and emission filter at 530/30. Single-color analysis was performed with CellQuest software (Becton Dickinson).

### RPC differentiation

For differentiation, retinal spheres were dissociated by the modified chemical dissociation method described above to obtain single-cell suspension. Cells were seeded at a concentration of 1×10^5^ cells/ml in 12 well  culture plates on glass coverslips coated with poly-D-lysine and laminin (Sigma), in basic medium in the absence of bFGF and EGF growth factors and supplemented with 5% FBS. The culture medium was changed every 2–3 days, and cells were allowed to differentiate for up to 2 weeks. RPCs were labeled by incubating with 10 μM BrdU (Sigma) in growth medium for 48 h and analyzed by immunocytochemistry.

### Immunocytochemistry

The expression patterns of various cell lineage markers were analyzed using the following antibodies: 1:100 dilution mouse anti-nestin mAb for undifferentiated precursors (Chemicon), 1:500 anti-MAP-2 (microtubule-associated protein 2) mAb for mature neurons (Chemicon), 1:100 anti-opsin mAb for photoreceptors (Sigma), 1:800 anti-GFAP (glial fibrillary acidic protein) mAb for glial cells (Chemicon), and 1:100 anti-BrdU mAb for proliferating cells with incorporated BrdU (Zymed Laboratories).

Cells were fixed in ice-cold 4% paraformaldehyde in PBS and washed twice with ice-cold PBS. Cells were permeabilized with 0.2% Triton-X, and nonspecific binding sites were blocked using 10% donkey serum. To label intracellular protein markers, we incubated cells with primary mAb in PBS/0.1% Triton-X for 1 h at room temperature, followed by 45 min incubation with rhodamine-conjugated donkey anti-mouse IgG antibody (Jackson ImmunoResearch, West Grove, PA). Nuclei were counterstained with 1 μg/ml 4',6-diamidino-2-phenylindole (DAPI). The coverslips were mounted onto glass slides using water-based Aqua-PolyMount media (Polyscience). Samples were analyzed by Leica TCS SP5 confocal microscopy with diode laser for excitation at 405 nm and emission at 480 nm for DAPI, argon laser excitation at 488 and emission at 510–570 nm for YFP, and HeNe laser excitation at 543 nm and emission at 600–680 nm for rhodamine. Alternatively, samples were analyzed by Zeiss Axiovert 200 m fluorescence microscope with a mercury 100 W fluorescence light source (Zeiss, Thornwood, NY), which had 47% of the radiant output from a Zeiss HBO 100 lamp lies between the wavelengths of 320 and 700 nm. The filter set for DAPI was excitation 365, beam splitter FT 395, and emission BP 445/50; the filter set for YFP was excitation BP 475/40, beam splitter FT 500, and emission 530/50; and the filter set for rhodamine was excitation BP 565/30, beam splitter FT 585, and emission BP 620/60. For each immunostaining, at least 100 cells were counted to determine the percentage of cells with positive signal.

## Results

### Long-term expression of YFP reporter in undifferentiated RPCs

**Figure 2:**
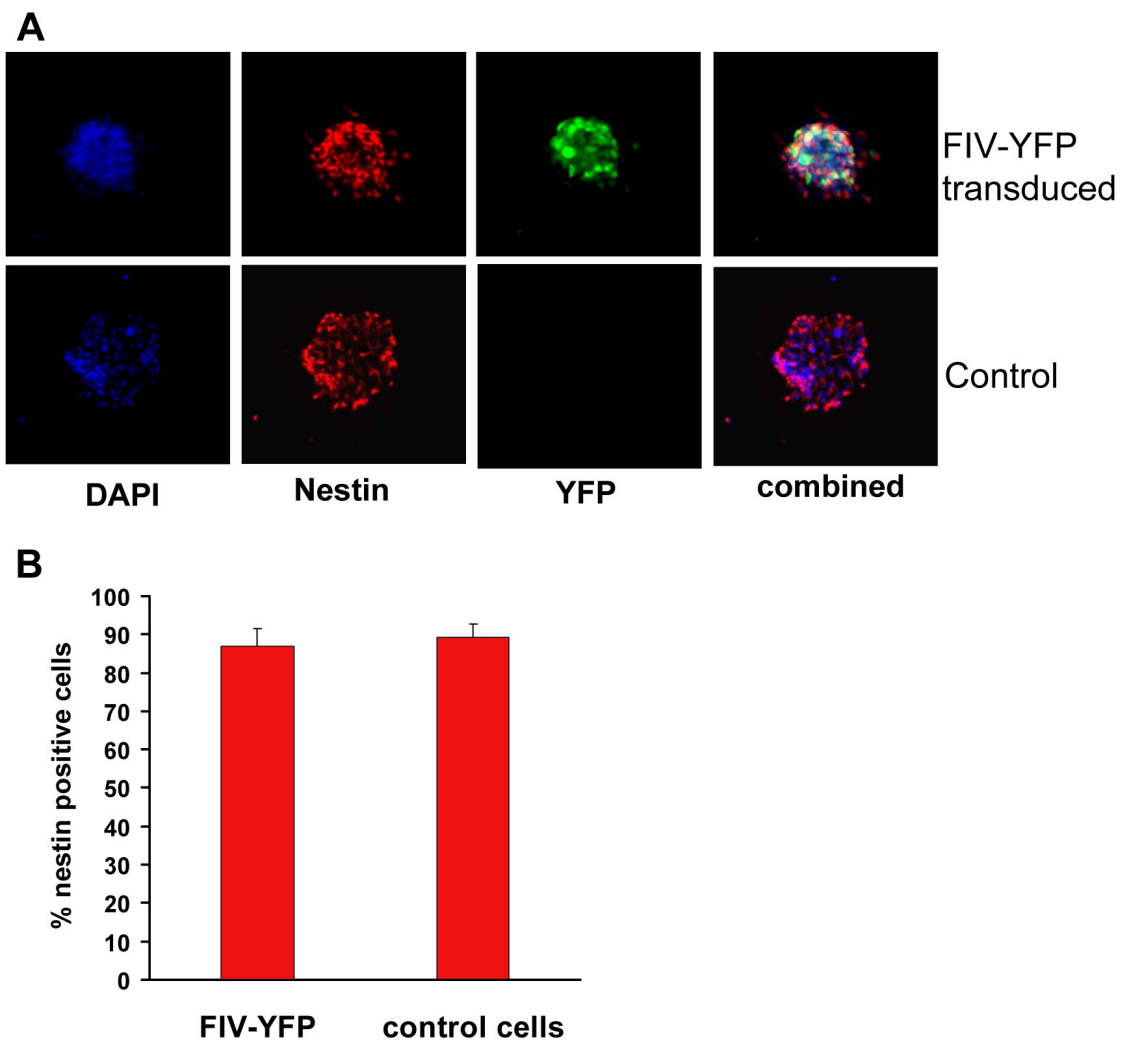
Nestin expression in FIV-transduced retinal progenitor cells. **A:** Immunocytochemistry for nestin expression (red signal) in retinal progenitor cell (RPC) spheres cultured under proliferating conditions. The nuclei of all cells were stained with DAPI (blue signal) and analyzed using a Zeiss Axiovert 200 m fluorescence microscope. **B:** Quantitative analysis of nestin-positive cells in feline immunodeficiency virus (FIV) for yellow fluorescent protein (YFP)-infected and noninfected RPCs. The percentage of nestin-expressing cells was determined by counting at least 100 cells in three independent experiments. Results are expressed as mean±SEM; p<0.05 (Student’s t-test; FIV-YFP versus control).

**Figure 3:**
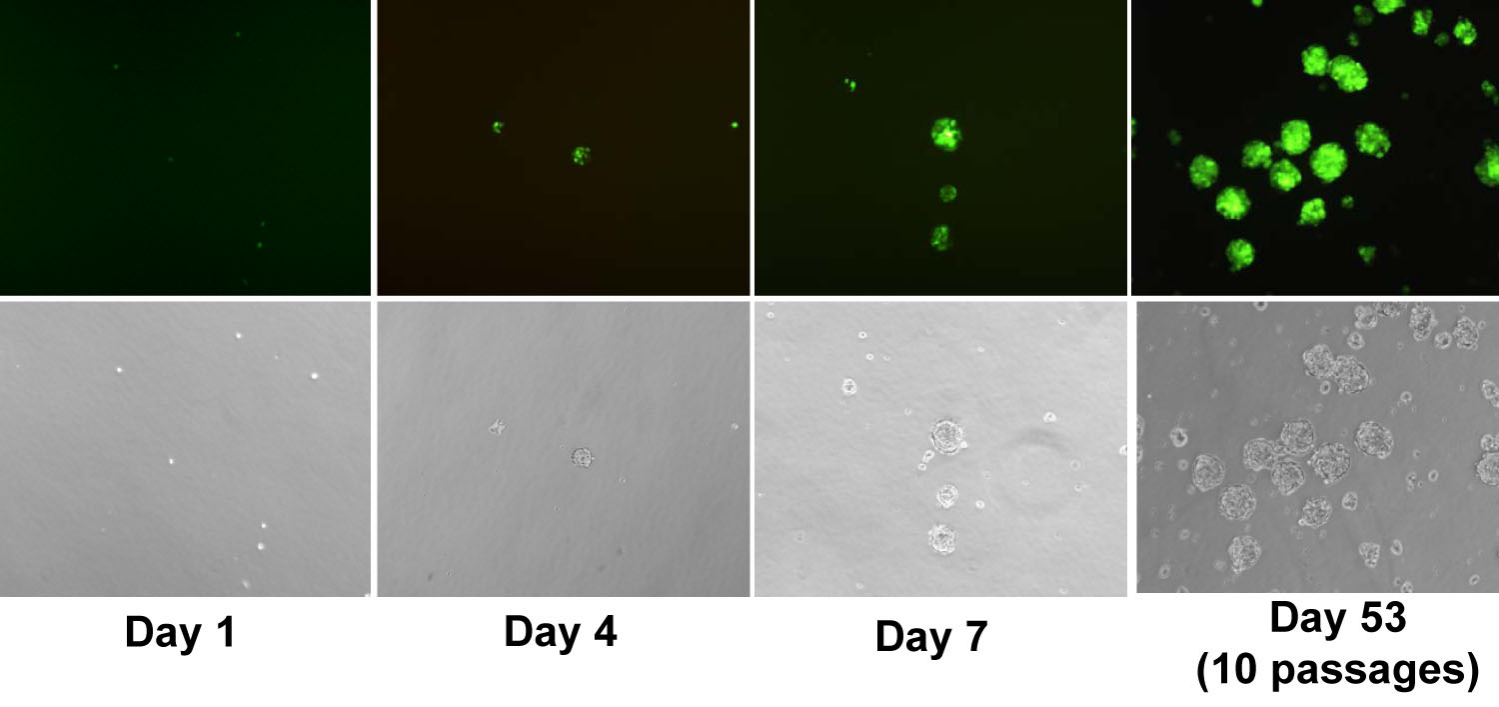
Long-term expression of YFP reporter in FIV-transduced RPCs. Dissociated RPCs were transduced with feline immunodeficiency virus (FIV) expressing yellow fluorescent protein (YFP) and plated at a low density of 10 cells/μl in the complete growth medium to generate clonal spheres. Cells were monitored, and images captured at days 1, 4, and 7 post-dissociation by the inverted fluorescence microscope to detect the presence of secondary RPC spheres. Long-term expression of YFP reporter was still detected after 10 passages. Shown are live-phase contrast cell images (fluorescence and bright field) from the representative culture.

**Figure 4:**
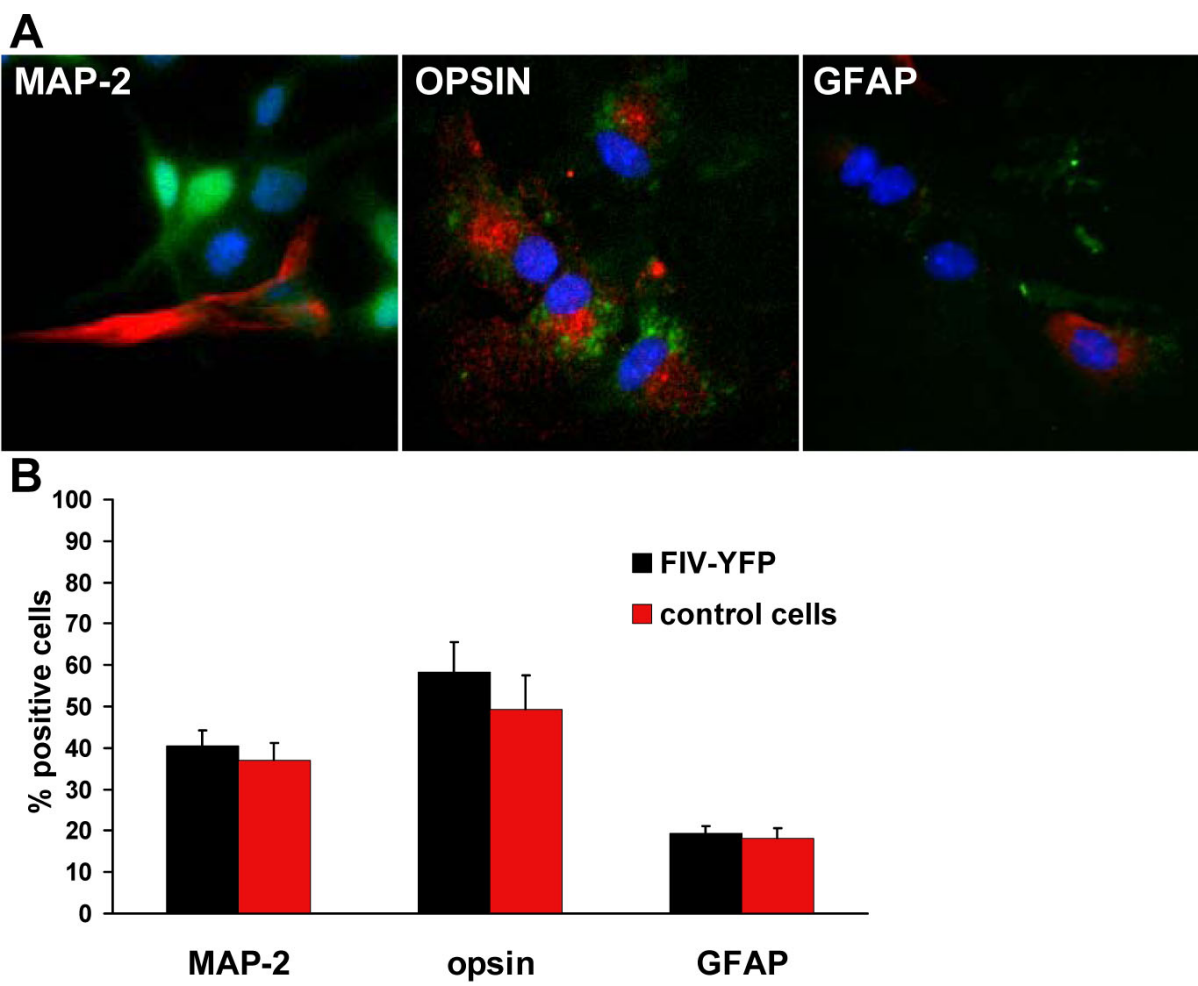
Multipotentiality. **A:** Retinal progenitor cells (RPCs) transduced with feline immunodeficiency virus (FIV) for yellow fluorescent protein (YFP) were cultured under differentiated conditions for 14 days to give rise to neuronal and glial cell types. Cells that stained positive for microtubule-associated protein 2 (MAP-2), opsin, and glial fibrillary acidic protein (GFAP) were analyzed by confocal microscopy, and signaling displayed in red. Green signal was YFP, and blue signal was DAPI staining for nuclei. **B:** Quantitative analysis of cells positive for MAP-2, opsin, and GFAP  in RPC group infected with or without FIV-YFP. At least 100 cells were counted from three independent experiments. Results are expressed as mean±SEM; p>0.05 (Student’s t-test; FIV-YFP versus control).

Cells isolated from mouse retina at postnatal day 1 were cultured as a monolayer in FBS-supplemented medium until they reached confluence. When cells were switched to FBS-free mitogen-containing medium, retinal spheres formed that showed positive staining for progenitor cell marker nestin (Figure 2). The RPCs exhibited clonogenic proliferation capacity (clonality; Figure 3) and the ability to give rise to multiple cell lineages (multipotentiality; Figure 4).

FIV is a nonprimate lentivirus capable of infecting both dividing and nondividing cells [13]. It is also able to integrate transgene of interest into the host chromosome for long-term expression. In the present study, FIV efficiently transduced high levels of YFP expression in more than 97.5% of RPCs (Figure 1). Sustained transgene expression of YFP reporter was detected up to 53 days and 10 passages in culture, with no sign of decrease in expression (Figure 3). FIV transduction neither altered the expression pattern of nestin (Figure 2), nor affected the differentiation capacity of RPCs to other cell lineages (Figure 4). Cell types generated by RPC differentiation were analyzed by immunocytochemistry for cell-specific marker expression, including MAP-2 for mature neurons, opsin for photoreceptors, and GFAP for glial cells (Figure 4A). No difference was observed in the percentage of cells positive for those markers between FIV-YFP transduced and control cells (Figure 4B). In addition, FIV transduction did not affect RPC growth rate (data not shown). These results indicated that FIV transduction induced strong and stable transgene expression without affecting RPC self-renewal and differentiation capacities.

### Loss of YFP reporter expression in differentiated RPCs

**Figure 5:**
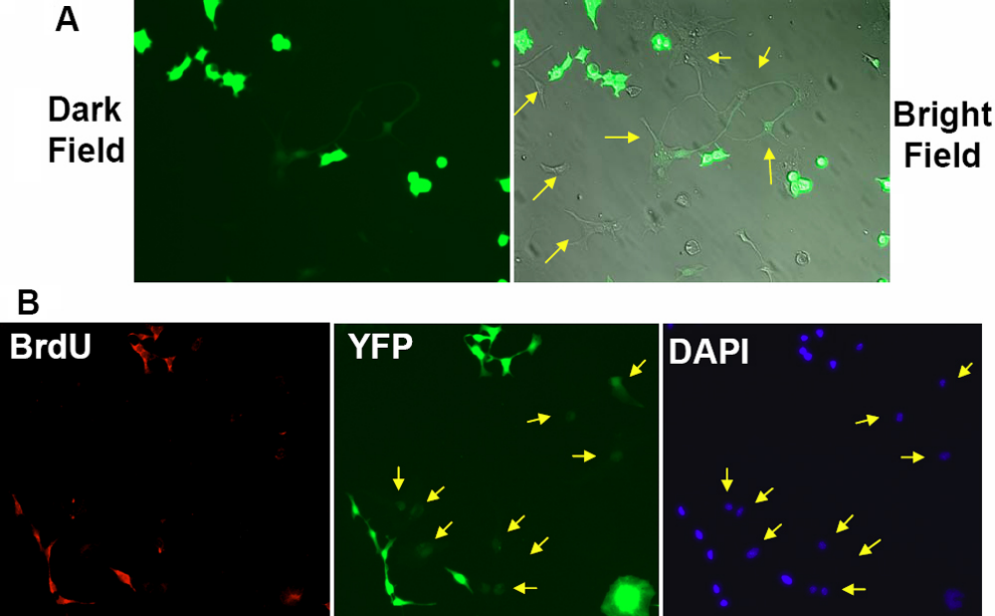
YFP expression in differentiated retinal progenitor cells. **A:** Live images of FIV-YFP infected cells cultured for 14 days under differentiated conditions from a representative experiment. The cells have adopted a variety of neuronal cell morphologies and have downregulated the expression of YFP (arrows). **B:** Immunocytochemistry of cells labeled with BrdU (red signal) and cultured under differentiating conditions. BrdU-negative cells downregulated the expression of YFP (arrows). The nuclei of all cells were stained with DAPI (blue signal). The results were examined using a Zeiss Axiovert fluorescence microscope, and images from the representative experiments are shown.

Because of a previous report of the loss of transgene expression in lentivirus- and retrovirus-transduced NPCs [14], it was important to analyze FIV-mediated transgene expression in differentiated RPCs. Downregulation of YFP expression was observed mostly in differentiated cells that exhibited attachment and extended cellular processes (Figure 5A). All the cells expressing a high level of YFP lacked extended cellular processes. During normal RPC differentiation, RPCs lose their self-renewal capacity and emerge as differentiated postmitotic retinal cell lineages, such as retinal ganglion cells, photoreceptors, and Müller glial cells. To delineate the correlation between cell proliferation capacity and transgene downregulation, we labeled proliferating cells with BrdU. The results revealed that BrdU-positive RPCs had a high level of YFP expression, while transgene expression was substantially downregulated in BrdU-negative cells (Figure 5B). These data suggested that the loss of YFP expression occurred only in differentiated postmitotic cells.

## Discussion

Gene transfer into multipotent progenitor cells is an integral part of ex vivo gene therapy, which has been shown to be able to successfully halt or delay the progression of neural degenerative diseases or to treat various pathological conditions [17,18]. Retroviral and lentiviral vectors have been widely used for ex vivo gene therapy because they are able to integrate into the host chromosomes for long-term gene expression [19]. It is anticipated that genetically engineered progenitor cells, after implantation, may differentiate toward distinct cell lineages to replace the degenerated cells. Stable expression of transgenes in undifferentiated progenitor cells, and—more important—in differentiated cells, represents an important prerequisite for successful ex vivo gene therapy.

Gene silencing is a general term encompassing several related phenomena. The most dramatic effect was reported about 30 years ago when murine leukemia virus (MLV) was subjected to complete transcriptional silencing after being introduced into embryonic carcinoma cells [20,21]. A less well known fact is that retrovirus vectors are also susceptible to "extinction", a term that refers to the progressive silencing of an initially expressed transgene during long-term culture or differentiation [22].

Retroviral silencing has been attributed to reduced transcriptional initiation at the promoter that can be caused by the binding of trans-acting factors to silencer elements in the viral long-terminal repeats (LTRs) [8,9]. Chromatin condensation caused by de novo cytosine methylation of CpG sequences located within LTRs may contribute to transgene silencing as well [23,24]. Histone deacetylation of the integrated transgenic DNA was suggested to be partially responsible for the decrease [9,25]. The construction of viral vectors lacking silencer elements, the inclusion of improved positive regulatory elements, and the use of internal promoters have been reported to improve duration of gene expression [8]. Inserting a copy of DNA insulator into LTRs of an HIV-based lentiviral vector can prevent enhancer-promoter interactions and protect transgenic expression cassettes from silencing and positional effects [26-28]. However, a previous study showed that an insulator was only able to partially protect against differentiation-dependent downregulation of HIV-based transgene expression in NPCs, both in vitro and in vivo, but not progressive transgene silencing in proliferating cells [29]. These results suggest that the mechanism for progressive silencing is different from the one for differentiation-dependent silencing. Our study revealed that although progressive loss of FIV-mediated transgene expression was not detected in undifferentiated RPCs, differentiation induced substantial loss in transgene expression. It has yet to be determined whether the insertion of an insulator in FIV LTR may protect against differentiation-dependent silencing in RPCs.

Transgene expression may also be downregulated by other mechanisms. For example, the  cytomegalovirus promoter has been shown to be vulnerable to silencing due to DNA methylation [30]. Previous investigations indicated that different cellular and viral promoters exhibited distinct and dynamic properties not only in terms of promoter strength but also with respect to differentiation stage-specific activity [31]. Thus, it is necessary to thoroughly examine and compare different promoters for optimal transgene expression in RPCs.

Other possible explanations for the loss of YFP signal may include changes in translation efficiency in a new cellular environment due to RPC differentiation or “dilution” of YFP signal in the differentiated RPCs with larger cellular areas. Although less likely, these possibilities cannot be completely eliminated.

RPCs share many similarities to NPCs. Both derive from neural tissues, grow as spheres in similar defined serum-free culture media, and are capable of giving rise to similar, but distinct, neural cell lineages. Although transgene silencing by MLV-based retroviral vector and HIV-based lentiviral vector has been well documented in NPCs, it has not been documented for FIV-based lentiviral vector. Because of the similarities between RPCs and NPCs, a previous report of transgene silencing in differentiated NPCs transduced by MLV-based retroviral vectors and HIV-based lentiviral vectors raised the possible concern of transgene silencing in RPCs [14]. Our findings indicated that FIV transduction induced long-term transgene expression in undifferentiated RPCs. Yet, differentiation of RPCs was accompanied by a rapid loss of YFP expression, indicating a close relationship between RPC differentiation and transgene silencing. The loss of YFP expression in differentiated RPCs was likely due to transcriptional silencing that was previously reported for retroviral and lentiviral vectors. Intriguingly, previous studies showed that direct in vivo injection of lentiviral vectors resulted in stable reporter expression in differentiated neurons and glia for up to 16 months [14]. Moreover, a recent study by Rompani and Cepko [32] showed that Moloney Murine Leukemia viral (MMLV) vector and HIV-based lentiviral vector efficiently transfected chick retinal progenitor cells or determined progenitor cells in ovo. The differentiated horizontal cell lineages remained reporter-positive. The discrepancy could be due to the uniqueness related to FIV, or it may have been caused by in vitro culture conditions, such as long-term culture after the FIV infection. Our results suggested that development of any future ex vivo gene therapy using RPCs, especially with lentiviral or retroviral vectors, should pay special consideration to transgene silencing not only in undifferentiated progenitors, but also in differentiated cells.
